# Ethyl 4-chloro-2′-fluoro-3-hy­droxy-5-methyl­biphenyl-2-carboxyl­ate

**DOI:** 10.1107/S160053681103217X

**Published:** 2011-08-17

**Authors:** Muhammad Adeel, Peter Langer, Alexander Villinger

**Affiliations:** a Gomal University, Department of Chemistry, Dera Ismail Khan (KPK), Pakistan; b Universität Rostock, Institut für Chemie, Albert-Einstein-Strasse 3a, 18059 Rostock, Germany; c Leibniz-Institut für Katalyse e.V., an der Universität Rostock, Albert-Einstein-Strasse 29a, 18059 Rostock, Germany

## Abstract

In the title compound, C_16_H_14_ClFO_3_, the dihedral angle between the mean planes of the two benzene rings is 71.50 (5)°. Due to an intra­molecular O—H⋯O hydrogen bond between the hy­droxy group and the carbonyl O atom of the ethyl ester group, the ethyl ester group lies within the ring plane. The crystal structure is consolidated by inter­molecular C—H⋯O and C—H⋯F inter­actions.

## Related literature

For a related structure, see: Adeel *et al.* (2009 ▶). For synthetic procedures and the pharmacological relevance of 3-chloro­salicylates, see: Wolf *et al.* (2009 ▶).
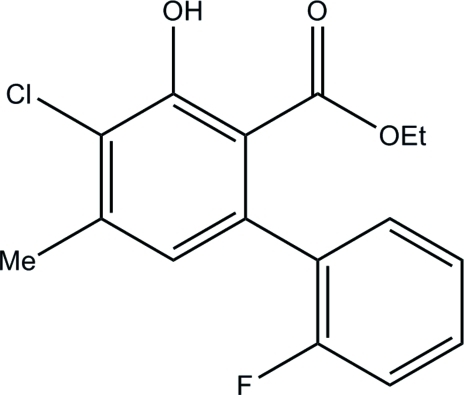


## Experimental

### 

#### Crystal data

C_16_H_14_ClFO_3_
*M* *_r_* = 308.73
Triclinic, 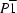

*a* = 8.212 (4) Å
*b* = 9.780 (3) Å
*c* = 10.156 (3) Å
α = 71.18 (3)°
β = 76.41 (2)°
γ = 71.34 (2)°
*V* = 723.5 (5) Å^3^
*Z* = 2
Mo *K*α radiation
μ = 0.28 mm^−1^
*T* = 173 K
0.31 × 0.18 × 0.08 mm

#### Data collection

Bruker APEXII KappaCCD diffractometer
Absorption correction: multi-scan (*SADABS*; Sheldrick, 2004 ▶) *T*_min_ = 0.918, *T*_max_ = 0.978
12813 measured reflections
3773 independent reflections
2902 reflections with *I* > 2σ(*I*)
*R*_int_ = 0.028

#### Refinement

*R*[*F* ^2^ > 2σ(*F* ^2^)] = 0.047
*wR*(*F* ^2^) = 0.139
*S* = 1.07
3773 reflections
195 parameters
H atoms treated by a mixture of independent and constrained refinement
Δρ_max_ = 0.87 e Å^−3^
Δρ_min_ = −0.24 e Å^−3^

### 

Data collection: *APEX2* (Bruker, 2003 ▶); cell refinement: *SAINT* (Bruker, 2003 ▶); data reduction: *SAINT*; program(s) used to solve structure: *SHELXS97* (Sheldrick, 2008 ▶); program(s) used to refine structure: *SHELXL97* (Sheldrick, 2008 ▶); molecular graphics: *ORTEP-3* (Farrugia, 1997 ▶); software used to prepare material for publication: *SHELXL97*.

## Supplementary Material

Crystal structure: contains datablock(s) I, global. DOI: 10.1107/S160053681103217X/pv2433sup1.cif

Structure factors: contains datablock(s) I. DOI: 10.1107/S160053681103217X/pv2433Isup2.hkl

Supplementary material file. DOI: 10.1107/S160053681103217X/pv2433Isup3.cml

Additional supplementary materials:  crystallographic information; 3D view; checkCIF report

## Figures and Tables

**Table 1:** Hydrogen-bond geometry (Å, °)

*D*—H⋯*A*	*D*—H	H⋯*A*	*D*⋯*A*	*D*—H⋯*A*
C7—H7*C*⋯O1^i^	0.98	2.59	3.473 (3)	150
C9—H9*A*⋯F^ii^	0.99	2.40	3.320 (3)	155
O1—H1⋯O2	0.82 (3)	1.77 (3)	2.521 (2)	153 (3)

Symmetry codes: (i) 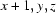; (ii) 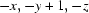.

**Fig. 1.**
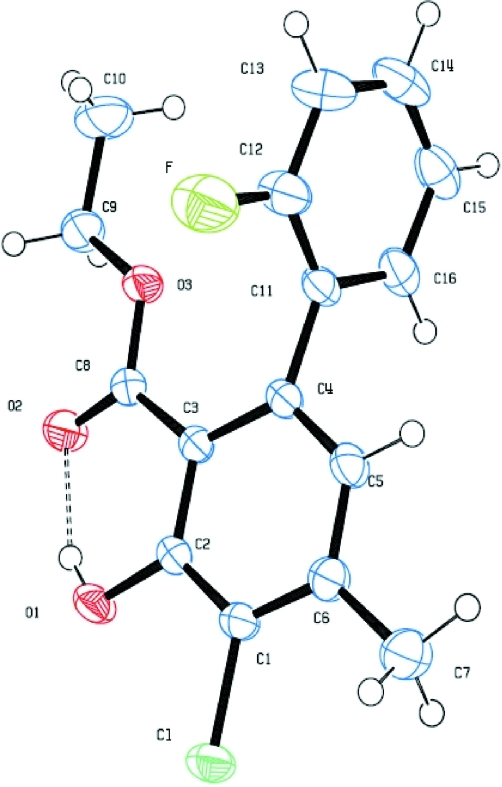
Molecular structure of the title compound drawn with thermal elliposids at the 50% probability level.

## supplementary crystallographic information

### Comment

Functionalized chloroarenes are of considerable pharmacological relevance. 3-Chlorosalicylates and related compounds are employed as pharmacological agents (Wolf *et al.*, 2009).

In the title compound (Fig. 1), the dihedral angle between the mean planes of the two benzene rings is 71.50 (5)°. The ethyllester group lies within the ring plane due to an intramolecular hydrogen bond between the hydroxyl group and the carbonyl O atom of the ehtyllester group, O1—H1···O2. The crystal structure is consolidated by weak C7—H7C···O1 and C9—H9A···F intermolecular interactions.

### Experimental

Experimental: The title compound was prepared according to a previously published procedure (Wolf *et al.* 2009). To a CH_2_Cl_2_ (6 ml) solution of 1-(2-fluoro-phenyl)-3-trimethylsilanyloxy-but-2-en-1-one (720 mg, 2.8 mmol) and 4-chloro-1-ethoxy-1,3-bis-trimethylsilanyloxy-buta-1,3-diene (970 mg, 3.1 mmol) was added TiCl_4_ (0.34 ml, 3.1 mmol) at 195 K under argon atmosphere. The solution was allowed to warm to ambient temperature within 20 hrs. To the solution was added a saturated solution of NaHCO_3_ (15 mL). The organic and the aqueous layers were separated and the latter was extracted with diethyl ether (3 × 20 ml). The filtrate was concentrated in vacuo and the residue was purified by chromatography (silica gel, EtOAc / n-heptane = 1:4). The title compound was isolated as a colorless crystalline solid. Yield: 390 mg, 45%. Mp. = 352 K. Crystallization from a saturated dichloromethane/methanol (9:1) solution at ambient temperature gave colorless crystals.

### Refinement

The H atom bonded to O1 was located from a difference map and refined freely. Other H atoms were positioned geometrically and refined using a riding model, with C—H = 0.98 (methyl groups) or 0.95 Å (aryl CH) and with *U*_iso_(H) = 1.5 times *U*_eq_(C) (methyl groups) or with *U*_iso_(H) = 1.2 times *U*_eq_(C) (aryl CH). Torsion angles of all methyl groups were allowed to refine.

### Crystal data

**Table:**

C_16_H_14_ClFO_3_	*Z* = 2
*M**_r_* = 308.73	*F*(000) = 320
Triclinic, *P*1	*D*_x_ = 1.417 Mg m^−^^3^
Hall symbol: -P 1	Melting point: 342 K
*a* = 8.212 (4) Å	Mo *K*α radiation, λ = 0.71073 Å
*b* = 9.780 (3) Å	Cell parameters from 12813 reflections
*c* = 10.156 (3) Å	θ = 0.9–1.0°
α = 71.18 (3)°	µ = 0.28 mm^−^^1^
β = 76.41 (2)°	*T* = 173 K
γ = 71.34 (2)°	Block, colourless
*V* = 723.5 (5) Å^3^	0.31 × 0.18 × 0.08 mm

### Data collection

**Table:**

Bruker APEXII KappaCCD diffractometer	3773 independent reflections
Radiation source: fine-focus sealed tube	2902 reflections with *I* > 2σ(*I*)
graphite	*R*_int_ = 0.028
ω scans	θ_max_ = 29.0°, θ_min_ = 4.6°
Absorption correction: multi-scan (*SADABS*; Sheldrick, 2004)	*h* = −11→11
*T*_min_ = 0.918, *T*_max_ = 0.978	*k* = −10→13
12813 measured reflections	*l* = −13→13

### Refinement

**Table:**

Refinement on *F*^2^	Primary atom site location: structure-invariant direct methods
Least-squares matrix: full	Secondary atom site location: difference Fourier map
*R*[*F*^2^ > 2σ(*F*^2^)] = 0.047	Hydrogen site location: inferred from neighbouring sites
*wR*(*F*^2^) = 0.139	H atoms treated by a mixture of independent and constrained refinement
*S* = 1.07	*w* = 1/[σ^2^(*F*_o_^2^) + (0.0762*P*)^2^ + 0.1732*P*] where *P* = (*F*_o_^2^ + 2*F*_c_^2^)/3
3773 reflections	(Δ/σ)_max_ < 0.001
195 parameters	Δρ_max_ = 0.87 e Å^−^^3^
0 restraints	Δρ_min_ = −0.24 e Å^−^^3^

### Special details

**Table:**

Experimental. Yield: 390 mg, 45%. Mp. = 352(???) K ^1^H NMR (250 MHz, CDCl_3_): δ = 0.75 (t,3*H*, J = 6.5 Hz, CH_3_) 2.34 (s, 3H, CH_3_), 3.96 (q, 2H, J = 7.0 Hz,OCH_2_),6.60 (s, 1H, ArH), 6.91–6.98 (m, 1H, ArH), 7.06–7.11 (m, 2H, ArH), 7.22 (m, 1H, ArH), 11.53 (s, 1 H, OH). ^13^C NMR (62 MHz, CDCl_3_): δ = 13.0 (CH3), 20.7 (CH3), 61.5 (OCH_2_), 111.0 (C), 114.4, 114.8 (CH), 122.3 (C), 123.7, 124.4, 129.0 (CH), 135.4, 142.9, 142.4, 157.7, 161.0 (C), 170.5 (C=O). IR (ATR, cm ^-1^): \~ν = 3040 (w), 2979 (*m*), 1657 (*m*), 1604 (*m*), 1495 (*m*), 1440 (*m*), 1374 (*s*), 1260 (*s*), 1215 (*s*), 759 (*s*). GC—MS (EI, 70 eV): m/*z* (%): 310 (*M*^+^, ^37^Cl, 8), 308 (*M*^+^, 24), 262 (100), 234 (12), 199 (11), 170 (24). HRMS (EI, 70 eV): calcd for C_116_H_14_ClFO_3_ [*M*, ^35^Cl]: 308.06100; found 308.060555.
Geometry. All s.u.'s (except the s.u. in the dihedral angle between two l.s. planes) are estimated using the full covariance matrix. The cell s.u.'s are taken into account individually in the estimation of s.u.'s in distances, angles and torsion angles; correlations between s.u.'s in cell parameters are only used when they are defined by crystal symmetry. An approximate (isotropic) treatment of cell s.u.'s is used for estimating s.u.'s involving l.s. planes.
Refinement. Refinement of *F*^2^ against ALL reflections. The weighted *R*-factor *wR* and goodness of fit *S* are based on *F*^2^, conventional *R*-factors *R* are based on *F*, with *F* set to zero for negative *F*^2^. The threshold expression of *F*^2^ > 2σ(*F*^2^) is used only for calculating *R*-factors(gt) *etc*. and is not relevant to the choice of reflections for refinement. *R*-factors based on *F*^2^ are statistically about twice as large as those based on *F*, and *R*- factors based on ALL data will be even larger.

### Fractional atomic coordinates and isotropic or equivalent isotropic displacement parameters (Å^2^)

**Table:**

	*x*	*y*	*z*	*U*_iso_*/*U*_eq_	
Cl	−0.02886 (6)	0.86275 (5)	0.43812 (5)	0.03926 (16)	
F	0.3361 (2)	0.44171 (16)	−0.01240 (13)	0.0725 (5)	
O1	−0.18805 (15)	0.68979 (15)	0.35763 (15)	0.0384 (3)	
H1	−0.225 (3)	0.630 (3)	0.341 (3)	0.058*	
O2	−0.19906 (16)	0.46568 (15)	0.29190 (16)	0.0422 (3)	
O3	0.04108 (15)	0.32871 (13)	0.19277 (14)	0.0341 (3)	
C1	0.0830 (2)	0.71258 (18)	0.36909 (17)	0.0280 (3)	
C2	−0.0136 (2)	0.63762 (17)	0.33593 (17)	0.0265 (3)	
C3	0.07182 (19)	0.51135 (17)	0.28378 (16)	0.0247 (3)	
C4	0.2551 (2)	0.46328 (17)	0.26719 (16)	0.0253 (3)	
C5	0.3454 (2)	0.54158 (19)	0.30155 (18)	0.0307 (4)	
H5	0.4687	0.5086	0.2899	0.037*	
C6	0.2626 (2)	0.66689 (19)	0.35253 (19)	0.0313 (4)	
C7	0.3663 (3)	0.7464 (3)	0.3907 (3)	0.0478 (5)	
H7A	0.3351	0.7412	0.4911	0.072*	
H7B	0.3410	0.8515	0.3355	0.072*	
H7C	0.4905	0.6983	0.3704	0.072*	
C8	−0.0406 (2)	0.43450 (18)	0.25693 (18)	0.0284 (3)	
C9	−0.0649 (3)	0.2461 (2)	0.1702 (2)	0.0431 (5)	
H9A	−0.1626	0.3160	0.1207	0.052*	
H9B	−0.1129	0.1862	0.2612	0.052*	
C10	0.0523 (3)	0.1462 (3)	0.0828 (3)	0.0612 (7)	
H10A	0.1556	0.0866	0.1276	0.092*	
H10B	0.0874	0.2072	−0.0110	0.092*	
H10C	−0.0092	0.0792	0.0744	0.092*	
C11	0.3638 (2)	0.32652 (18)	0.22316 (18)	0.0280 (3)	
C12	0.4056 (3)	0.3214 (2)	0.0855 (2)	0.0422 (4)	
C13	0.5149 (3)	0.1975 (3)	0.0445 (2)	0.0536 (6)	
H13	0.5400	0.1975	−0.0517	0.064*	
C14	0.5866 (3)	0.0741 (2)	0.1454 (3)	0.0487 (5)	
H14	0.6625	−0.0120	0.1188	0.058*	
C15	0.5496 (2)	0.0742 (2)	0.2836 (2)	0.0428 (5)	
H15	0.5994	−0.0118	0.3529	0.051*	
C16	0.4392 (2)	0.19994 (19)	0.3228 (2)	0.0350 (4)	
H16	0.4147	0.1996	0.4191	0.042*	

### Atomic displacement parameters (Å^2^)

**Table:**

	*U*^11^	*U*^22^	*U*^33^	*U*^12^	*U*^13^	*U*^23^
Cl	0.0353 (2)	0.0323 (2)	0.0569 (3)	−0.00351 (17)	−0.00615 (19)	−0.0267 (2)
F	0.0975 (11)	0.0589 (8)	0.0352 (7)	0.0110 (8)	−0.0107 (7)	−0.0092 (6)
O1	0.0198 (6)	0.0397 (7)	0.0629 (9)	−0.0002 (5)	−0.0055 (5)	−0.0315 (7)
O2	0.0243 (6)	0.0488 (8)	0.0664 (9)	−0.0072 (5)	−0.0058 (6)	−0.0358 (7)
O3	0.0293 (6)	0.0323 (6)	0.0484 (7)	−0.0034 (5)	−0.0091 (5)	−0.0233 (5)
C1	0.0259 (8)	0.0253 (7)	0.0348 (8)	−0.0036 (6)	−0.0039 (6)	−0.0141 (6)
C2	0.0217 (7)	0.0263 (7)	0.0319 (8)	−0.0022 (6)	−0.0055 (6)	−0.0115 (6)
C3	0.0217 (7)	0.0241 (7)	0.0295 (8)	−0.0038 (6)	−0.0048 (6)	−0.0102 (6)
C4	0.0232 (7)	0.0240 (7)	0.0260 (7)	−0.0028 (6)	−0.0027 (6)	−0.0072 (6)
C5	0.0198 (7)	0.0328 (8)	0.0405 (9)	−0.0054 (6)	−0.0026 (6)	−0.0142 (7)
C6	0.0266 (8)	0.0323 (8)	0.0392 (9)	−0.0094 (7)	−0.0041 (7)	−0.0140 (7)
C7	0.0310 (9)	0.0527 (12)	0.0750 (14)	−0.0154 (9)	−0.0031 (9)	−0.0364 (11)
C8	0.0269 (8)	0.0276 (8)	0.0335 (8)	−0.0031 (6)	−0.0085 (6)	−0.0130 (6)
C9	0.0378 (10)	0.0391 (10)	0.0661 (13)	−0.0065 (8)	−0.0155 (9)	−0.0304 (10)
C10	0.0624 (15)	0.0610 (15)	0.0806 (17)	−0.0138 (12)	−0.0091 (13)	−0.0497 (14)
C11	0.0201 (7)	0.0268 (8)	0.0371 (9)	−0.0038 (6)	−0.0011 (6)	−0.0130 (7)
C12	0.0457 (11)	0.0361 (10)	0.0382 (10)	−0.0026 (8)	0.0002 (8)	−0.0139 (8)
C13	0.0567 (13)	0.0537 (13)	0.0479 (12)	−0.0089 (10)	0.0120 (10)	−0.0298 (10)
C14	0.0357 (10)	0.0320 (10)	0.0755 (15)	−0.0042 (8)	0.0070 (10)	−0.0262 (10)
C15	0.0321 (9)	0.0269 (9)	0.0646 (13)	−0.0034 (7)	−0.0078 (9)	−0.0098 (8)
C16	0.0255 (8)	0.0288 (8)	0.0503 (10)	−0.0058 (6)	−0.0062 (7)	−0.0109 (7)

### Geometric parameters (Å, °)

**Table:**

Cl—C1	1.7294 (17)	C7—H7B	0.9800
F—C12	1.334 (2)	C7—H7C	0.9800
O1—C2	1.348 (2)	C9—C10	1.492 (3)
O1—H1	0.82 (3)	C9—H9A	0.9900
O2—C8	1.229 (2)	C9—H9B	0.9900
O3—C8	1.315 (2)	C10—H10A	0.9800
O3—C9	1.458 (2)	C10—H10B	0.9800
C1—C6	1.384 (2)	C10—H10C	0.9800
C1—C2	1.392 (2)	C11—C12	1.372 (3)
C2—C3	1.411 (2)	C11—C16	1.392 (3)
C3—C4	1.412 (2)	C12—C13	1.379 (3)
C3—C8	1.479 (2)	C13—C14	1.372 (3)
C4—C5	1.384 (2)	C13—H13	0.9500
C4—C11	1.490 (2)	C14—C15	1.365 (3)
C5—C6	1.394 (2)	C14—H14	0.9500
C5—H5	0.9500	C15—C16	1.388 (3)
C6—C7	1.501 (2)	C15—H15	0.9500
C7—H7A	0.9800	C16—H16	0.9500
			
C2—O1—H1	105.4 (18)	O3—C9—H9A	110.5
C8—O3—C9	116.61 (14)	C10—C9—H9A	110.5
C6—C1—C2	121.84 (15)	O3—C9—H9B	110.5
C6—C1—Cl	120.26 (13)	C10—C9—H9B	110.5
C2—C1—Cl	117.86 (12)	H9A—C9—H9B	108.7
O1—C2—C1	117.25 (14)	C9—C10—H10A	109.5
O1—C2—C3	122.85 (15)	C9—C10—H10B	109.5
C1—C2—C3	119.89 (14)	H10A—C10—H10B	109.5
C2—C3—C4	118.74 (14)	C9—C10—H10C	109.5
C2—C3—C8	116.40 (14)	H10A—C10—H10C	109.5
C4—C3—C8	124.79 (14)	H10B—C10—H10C	109.5
C5—C4—C3	119.21 (14)	C12—C11—C16	116.99 (16)
C5—C4—C11	115.36 (14)	C12—C11—C4	123.15 (16)
C3—C4—C11	125.31 (14)	C16—C11—C4	119.67 (15)
C4—C5—C6	122.64 (15)	F—C12—C11	118.25 (17)
C4—C5—H5	118.7	F—C12—C13	118.85 (19)
C6—C5—H5	118.7	C11—C12—C13	122.90 (19)
C1—C6—C5	117.68 (15)	C14—C13—C12	118.7 (2)
C1—C6—C7	121.70 (16)	C14—C13—H13	120.6
C5—C6—C7	120.60 (16)	C12—C13—H13	120.6
C6—C7—H7A	109.5	C15—C14—C13	120.53 (18)
C6—C7—H7B	109.5	C15—C14—H14	119.7
H7A—C7—H7B	109.5	C13—C14—H14	119.7
C6—C7—H7C	109.5	C14—C15—C16	119.91 (19)
H7A—C7—H7C	109.5	C14—C15—H15	120.0
H7B—C7—H7C	109.5	C16—C15—H15	120.0
O2—C8—O3	121.77 (15)	C15—C16—C11	120.95 (19)
O2—C8—C3	123.07 (15)	C15—C16—H16	119.5
O3—C8—C3	115.17 (14)	C11—C16—H16	119.5
O3—C9—C10	106.34 (17)		
			
C6—C1—C2—O1	179.12 (16)	C9—O3—C8—C3	177.37 (15)
Cl—C1—C2—O1	1.3 (2)	C2—C3—C8—O2	−8.1 (2)
C6—C1—C2—C3	0.0 (3)	C4—C3—C8—O2	168.84 (16)
Cl—C1—C2—C3	−177.88 (12)	C2—C3—C8—O3	172.18 (14)
O1—C2—C3—C4	−178.74 (15)	C4—C3—C8—O3	−10.9 (2)
C1—C2—C3—C4	0.4 (2)	C8—O3—C9—C10	173.83 (17)
O1—C2—C3—C8	−1.6 (2)	C5—C4—C11—C12	−102.9 (2)
C1—C2—C3—C8	177.49 (14)	C3—C4—C11—C12	81.1 (2)
C2—C3—C4—C5	−0.4 (2)	C5—C4—C11—C16	72.0 (2)
C8—C3—C4—C5	−177.26 (15)	C3—C4—C11—C16	−104.1 (2)
C2—C3—C4—C11	175.50 (15)	C16—C11—C12—F	−179.55 (18)
C8—C3—C4—C11	−1.4 (3)	C4—C11—C12—F	−4.6 (3)
C3—C4—C5—C6	0.1 (3)	C16—C11—C12—C13	1.0 (3)
C11—C4—C5—C6	−176.19 (15)	C4—C11—C12—C13	175.94 (19)
C2—C1—C6—C5	−0.3 (3)	F—C12—C13—C14	179.8 (2)
Cl—C1—C6—C5	177.53 (13)	C11—C12—C13—C14	−0.7 (4)
C2—C1—C6—C7	−178.94 (18)	C12—C13—C14—C15	0.3 (3)
Cl—C1—C6—C7	−1.2 (3)	C13—C14—C15—C16	−0.2 (3)
C4—C5—C6—C1	0.2 (3)	C14—C15—C16—C11	0.5 (3)
C4—C5—C6—C7	178.92 (18)	C12—C11—C16—C15	−0.9 (3)
C9—O3—C8—O2	−2.4 (3)	C4—C11—C16—C15	−176.02 (16)

### Hydrogen-bond geometry (Å, °)

**Table:**

*D*—H···*A*	*D*—H	H···*A*	*D*···*A*	*D*—H···*A*
C7—H7C···O1^i^	0.98	2.59	3.473 (3)	150
C9—H9A···F^ii^	0.99	2.40	3.320 (3)	155
O1—H1···O2	0.82 (3)	1.77 (3)	2.521 (2)	153 (3)

Symmetry codes: (i) *x*+1, *y*, *z*; (ii) −*x*, −*y*+1, −*z*.
